# Nanomanaging Chronic Wounds with Targeted Exosome Therapeutics

**DOI:** 10.3390/pharmaceutics17030366

**Published:** 2025-03-13

**Authors:** Anita Yadav, Anu Sharma, Mohini Moulick, Subhadip Ghatak

**Affiliations:** McGowan Institute for Regenerative Medicine, Department of Surgery, University of Pittsburgh, Pittsburgh, PA 15219, USA; any102@pitt.edu (A.Y.); ans831@pitt.edu (A.S.); mom186@pitt.edu (M.M.)

**Keywords:** functional wound closure, engineered exosomes, cell–cell communication, biocompatible scaffolds, exosome-targeted therapies

## Abstract

Chronic wounds pose a significant healthcare challenge, impacting millions of patients worldwide and burdening healthcare systems substantially. These wounds often occur as comorbidities and are prone to infections. Such infections hinder the healing process, complicating clinical management and proving recalcitrant to therapy. The environment within the wound itself poses challenges such as lack of oxygen, restricted blood flow, oxidative stress, ongoing inflammation, and bacterial presence. Traditional systemic treatment for such chronic peripheral wounds may not be effective due to inadequate blood supply, resulting in unintended side effects. Furthermore, topical applications are often impervious to persistent biofilm infections. A growing clinical concern is the lack of effective therapeutic modalities for treating chronic wounds. Additionally, the chemically harsh wound microenvironment can reduce the effectiveness of treatments, highlighting the need for drug delivery systems that can deliver therapies precisely where needed with optimal dosages. Compared to cell-based therapies, exosome-based therapies offer distinct advantages as a cell-free approach for chronic wound treatment. Exosomes are of endosomal origin and enable cell-to-cell communications, and they possess benefits, including biocompatibility and decreased immunogenicity, making them ideal vehicles for efficient targeting and minimizing off-target damage. However, exosomes are rapidly cleared from the body, making it difficult to maintain optimal therapeutic concentrations at wound sites. The hydrogel-based approach and development of biocompatible scaffolds for exosome-based therapies can be beneficial for sustained release and prolong the presence of these therapeutic exosomes at chronic wound sites. Engineered exosomes have been shown to possess stability and effectiveness in promoting wound healing compared to their unmodified counterparts. Significant progress has been made in this field, but further research is essential to unlock their clinical potential. This review seeks to explore the benefits and opportunities of exosome-based therapies in chronic wounds, ensuring sustained efficacy and precise delivery despite the obstacles posed by the wound environment.

## 1. Introduction

Chronic wounds are defined as those not progressing towards healing within four weeks despite the standard of care and are characterized by a hyperproliferative epidermal edge and poorly vascularized vessels [1,2,3,4]. Such wounds lead to chronic pain, loss of function, increased psychosocial stress, depression, prolonged hospitalization, financial burden, and increased morbidity and mortality [5,6]. The burden of chronic wounds presents a substantial challenge to the economy and global health [7,8,9,10,11,12]. According to retrospective research, around 8.2 million Medicare patients suffered wounds or related infections, costing between USD 28.1 billion and USD 96.8 billion annually [7,13]. Approximately three percent of the population over 65 years have open wounds that are often complicated by comorbidities, such as diabetes and obesity, by impairing oxygen delivery and collagen synthesis, which are essential for tissue repair [14,15,16]. Such wounds are difficult to manage and significantly increase healthcare costs [7]. The highest expenses are associated with surgical wounds and diabetic foot ulcers, with outpatient treatments incurring higher costs than inpatient treatments [17]. The economic impact of wounds is significant, with the wound care products market projected to reach USD 15–22 billion by the end of 2024 [7]. Despite this, funding for wound care research remains disproportionately low, underscoring the need for increased investment in wound care education and research to address the rising prevalence and cost of chronic wounds [7].

Education and training in wound care are often inadequate among healthcare professionals, further complicating effective management [18,19]. Additionally, scarring and fibrosis from wounds impose esthetic, functional, and psychological burdens on patients [20,21]. To address these challenges and overcome the shortcomings of conventional therapies, comprehensive and standardized education for physicians and nurses, coupled with advancements in therapeutic strategies, is essential. This review highlights the importance of cell-specific exosomes in effectively treating chronic wounds. Strategies such as advanced dressings, surface modifications of bioengineered exosomes, utilizing self-identification signals, and bypassing the mononuclear phagocyte system (MPS) will be some of the critical steps in formulating more patient-focused care for treating wounds chronicity.

## 2. Roads Leading to Wound Chronicity

Understanding the key contributing factors for chronic wound healing is crucial for effective management of chronic wounds [22]. Infection plays a significant role in wound chronicity. Bacteria in the wound produce toxins like Lipopolysaccharides (LPS) and Pyocyanin [23] and inflammatory mediators such as Interleukin-1 beta (IL-1β) and Tumor Necrosis Factor-alpha (TNF-α), that prolong the inflammatory phase, prevent re-epithelialization, and lead to necrotic tissue leading to bacterial growth [24,25]. The presence of foreign bodies, such as nonabsorbable sutures, is an ongoing source of infection, making it difficult for the wound to heal without surgical intervention.

Ischemia is another major contributing factor that results from reduced blood flow, such as arterial insufficiency (restricted oxygen and nutrient delivery), venous hypertension (increased pressure leads to capillary leak and hypoxia), and pressure injuries [26].

Metabolic conditions developed due to diabetes impair sensory perception, and motor function, leading to unnoticed injuries and abnormal pressure. It can also promote atherosclerosis, further compromising blood flow and healing capacity [27,28,29].

Immunosuppression from corticosteroids, immunosuppressive agents, and chemotherapeutic drugs significantly impacts wound healing [30]. Corticosteroids and chemotherapeutic agents interfere with wound healing by restricting fibroblast proliferation, disrupting angiogenesis, and impairing collagen synthesis and remodeling [31].

Radiation therapies break cellular deoxyribonucleic acid (DNA) strands and generate free radicals, leading to cell death, particularly in rapidly dividing cells [32,33]. Additionally, radiation causes vascular damage, leading to luminal thrombosis, reduced blood flow, and tissue necrosis [34].

## 3. Challenges and Management of Wound Care: The Need to Bridge Socioeconomic Gaps for Better Outcomes

The diversity in wound care standards throughout the United States resembles a mosaic of practices, with each element symbolizing an approach influenced by institutional and individual factors. Chronic wounds are marked by a paucity of myofibroblasts and a significant inflammatory infiltrate, predominantly neutrophils. They exhibit many pro-inflammatory cytokines, proteases, senescent cells, and reactive oxygen species (ROS), alongside persistent infection [1,35,36]. Repeated tissue injury instigates a pro-inflammatory cytokine cascade triggered by microbes and platelet-derived factors, including transforming growth factor-beta (TGF-β) and extracellular matrix (ECM) fragment molecules [1]. In chronic wounds, protease levels exceed inhibitors, causing ECM degradation and the breakdown of growth factors and receptors [1,35]. Keratinocytes at the wound margin express a gene signature indicative of partial proliferative activation [37], while fibroblasts in ulcerated wounds are senescent, exhibit reduced migratory capacity, and are unresponsive to TGF-β, leading to diminished levels of transforming growth factor-beta receptor (TGFβR) and its downstream components [38,39].

Open skin wounds are rapidly colonized by bacteria from the patient’s normal flora or contaminated external sources [7,40]. Biofilms formed by polymicrobial consortia impede healing and produce defective functional wound closure driven by microRNAs that destabilize junctional proteins required for barrier formation post-closure [41,42,43,44,45,46,47,48]. Ongoing patient-based studies, including diabetic foot ulcers, indicate that wounds lacking barrier function are prone to recurrence [49,50]. The treatment of chronic wounds is fraught with numerous challenges that stem from both intrinsic and extrinsic factors affecting wound healing processes, and also intensive follow-up and recurrence prevention methods like protective footwear and regular foot examination are essential [1,51]. Excessive and prolonged inflammation leads to ECM degradation [52,53], the presence of senescent cells causes impaired proliferative and secretory capacities [1]. The underlying pathologies contributing to chronic wounds require a multifaceted approach to specific types of wounds and their underlying causes [1,7].

An in-depth examination of the current literature on the management and evaluation of non-healing wounds highlights their substantial physiological and psychological challenges. The T.I.M.E. (Tissue, Inflammation/Infection, Moisture, Edge) framework for effectively assessing and managing chronic wounds is critical. Principals focus must be on the need for tissue debridement, control of inflammation and infection, maintenance of moisture balance, and advancement to facilitate wound healing [54,55]. A well-known triangle of wound assessment (TWA) includes three key areas—peri-wound skin, wound bed, and wound edge in the treatment regimen [56,57]. The effective and systematic management [27] of chronic wounds requires thorough patient assessment (includes detailed medical history via diagnostic tests and physical examination) [1,58] and a multidisciplinary approach [1,8,59,60].

The diversity in wound care standards resembles a mosaic of practices, with each element symbolizing an approach influenced by institutional and individual factors, resulting in the caliber and availability of wound care services, depending on the individual’s health, income sources, and social interactions [61,62]. For example, older patients heal slower while younger patients tend to heal quickly, people with low incomes or no insurance face obstacles in receiving care, and positive social connections lead to better healing of an individual [62,63,64,65]. An important study in this field is the “Geographical Genomics” research conducted by Idaghdour et al., which found that 50% of variations in gene activity are influenced by whether individuals live in urban areas, compared to 5% due to genetic factors [66]. Diagnosis should systematically assess systemic, regional, and local factors [67,68]. Effective management strategies must decrease healing duration through a combination of infection control, revascularization, metabolic control, minimization of immunosuppressive effects, and implementation of evidence-based patient-centered practices along with a cost-saving approach [12,62,69,70,71,72].

## 4. Need for New and Integrated Therapies

The heterogeneous and chemically loud micro-environment of chronic wounds limits the regenerative properties of materials. Contemporary techniques and wound therapies are insufficient for all stages of wound healing and predominantly concentrate on wound management rather than the actual healing of chronic wounds [60,73]. Most preclinical studies involve small animals such as rats, mice, and rabbits. These animals exhibit considerable differences in skin morphology and wound healing processes compared to humans, including variations in skin thickness, blood flow, and the presence of growth receptors. Notably, murine skin heals through contraction, while human skin heals via re-epithelialization [74]. In contrast, larger animals, such as pigs and monkeys, display more similarities to human skin, including a thick epidermis and well-developed dermal papillary bodies, and heal through re-epithelialization rather than contraction [75]. Despite this, studies on large animals are not widely utilized, limiting the translatability of findings due to the functional and metabolic differences between animal models and human systems [19,76].

The increasing number of patients with either hyperhealing or hypohealing wounds underscores the inefficacy of present wound-healing treatments due to the complex microenvironment characterized by hypoxia, inflammation, hyperglycemia, and infection [77]. Current therapeutic modalities encompass dressing changes, debridement, infection control, skin tissue transplantation, ECM application, mesenchymal stem cell (MSC) therapy, and negative pressure wound therapy (NPWT) [78,79,80]. Despite the advancements in these approaches, they face limitations such as complications, wound recurrence, and variability in healing efficacy [81]. This highlights a critical need for alternative therapeutic strategies that address these underlying pathological conditions to enhance chronic wound healing outcomes.

### 4.1. Commercial Products and Advanced Biomaterials

Commercial products, including biomaterials for chronic wound healing, primarily target symptoms such as moisture balance, scarring, fluid exudation, pressure relief, and infection [60]. Advanced biomaterials that mimic the ECM are typically more biocompatible, modulate immune response to resolve inflammation, reduce the risk of immune rejection, promote angiogenesis, support the dynamic environment of a healing wound, accommodate movement, and reduce the risk of wound reopening. Extracellular matrix membrane mimicking materials can be engineered to deliver drugs, such as antibiotics or anti-inflammatory agents, directly to the wound site, reducing the risk of systemic side effects and promoting regenerative capacity [60]. Several studies have focused on controlling cell behavior [82,83,84] and stimuli-responsive release, which can be triggered by the skin’s pH (ranges from pH 4 to pH 6) [85,86] or by exploiting temperature differences within the body to induce vasodilation and enhance nutrient and oxygen supply [60].

Hydrogel-based delivery systems offer a moist wound-healing environment and the sustained and stimuli-responsive release of therapeutics [87]. Commercially available hydrogels exhibit antibacterial, hemostatic, tissue adhesion, anti-ultraviolet, injectability, and self-healing properties, addressing limitations associated with systemic administration [88]. Several studies discovered that the hydrogel could eliminate accumulated reactive oxygen species (ROS), induce macrophage polarization toward the M2 phenotype, reduce excessive inflammation, and promote proliferation, re-epithelialization, collagen deposition, and the formation of new blood vessels [88,89]. The wound healing capacity of hydrogels can be boosted synergistically by including active biomolecules or cells [90,91].

### 4.2. Nanotherapeutic Approaches in Wound Healing

A nanotherapeutic-based approach has shown considerable promise in promoting wound healing with minimum scarring [92]. Various nanomaterial-based strategies such as nanofibers, nanogel, micelles, liposomes, polymeric, and inorganic or lipid nanoparticles have demonstrated significant potential to enhance cellular interaction, antimicrobial activity, and improved mechanical properties during the healing process (Table 1) [93,94]. For example, the integration of collagen with metallic nanoparticles such as silver, copper, zinc, and gold has emerged as a promising strategy in the design of multifunctional wound dressings [95]. Furthermore, modifications of these nanoparticle surfaces have shown considerable promise in enhancing therapeutic outcomes. For instance, Kelestemur et al. demonstrated that functionalizing silver nanoparticles with thiolated oligonucleotides prolonged the release of silver ions [96]. This controlled release mechanism could potentially reduce the frequency of application and improve patient compliance. Similarly, zinc oxide hydrogel bandages have been reported to absorb wound exudates efficiently while activating platelets and promoting coagulation, thereby facilitating a more rapid initial hemostatic response [97]. Other nanoparticles, such as graphene oxide and iron oxides, have also been used in wound healing [98]. Further advances include the development of metal–organic frameworks (MOFs) loaded with folic acid, as reported by Xiao et al. [99]. These MOFs provide a sustained release of Cu^2+^ ions, which in turn promote collagen synthesis, angiogenesis, and re-epithelialization, key processes in wound repair [99]. In a parallel approach, Jiang et al. engineered spaced-oriented scaffolds designed for silicon ion release, leveraging silicon-doped amorphous calcium phosphate nanoparticles [100]. Silicon ions, in turn, have been associated with enhanced angiogenesis and re-epithelialization in chronic wounds.

Despite these promising findings, several concerns remain. Silver-based nanomaterials, still suffers drawbacks. Reports of skin discoloration and the development of silver-resistant bacterial strains raise critical questions about the long-term safety and efficacy of these formulations [118]. Moreover, while alternative nanoparticles such as graphene oxide and iron oxides have been explored, their effectiveness in chronic wound healing remains under-investigated, warranting further rigorous studies [98]. The introduction of nitric oxide-doped PLGA nanoparticles has also shown potential in clearing methicillin-resistant *Staphylococcus aureus* (MRSA) biofilms, yet the clinical translation of these findings may be hindered by issues related to nanoparticle stability, dosage control, and potential cytotoxicity [98,119,120].

Nanomaterials interact directly with wound tissue and can potentially cause skin irritation or allergic reactions due to their unique characteristics, including size, stability, concentration, and shape [19,121,122,123]. Another critical factor is the controlled and sustained release of loaded drugs. The mechanisms governing drug release from nanomaterials are complex and not yet fully understood, posing a challenge to achieving consistent therapeutic outcomes [124]. Additionally, the cost of production is also a limiting factor, necessitating significant optimization to reduce expenses and make these therapies more accessible. In summary, while the integration of metallic nanoparticles with collagen-based wound dressings offers a multifaceted approach to tackling both infection and impaired wound healing, the field is still at a crossroads. Future research should focus on optimizing nanoparticle formulations to maximize their therapeutic benefits while mitigating safety risks, ensuring a balanced and effective approach to advanced wound care.

### 4.3. Exosome-Based Strategies

Exosomes are defined as small extracellular vesicles (sEVs) of endosomal origin, secreted by almost all cell types, having diameters ranging from 30 to 150 nm. It is important to acknowledge that the terms “exosome” and “small EV” are not synonymous. The development of exosome-based wound healing strategies is desired to understand underlying mechanisms and cellular cascades better [125,126,127,128]. These therapies are less likely to provoke an immune response, hence reducing the risk of rejection, and exosomes do not proliferate, unlike transplanted cells that might proliferate uncontrollably, leading to complications such as fibrosis or inappropriate tissue formation. Due to their small size, exosomes can penetrate deeper into tissues and target cells more effectively, allowing more efficient delivery of therapeutic molecules to the wound site. However, a few studies have mentioned several critical challenges, such as the potential degradation or altered functionality of exosomes in the inflammatory wound environment. Exosome production must be scaled up in a controlled environment to meet clinical needs, making therapies more cost-effective. Large-scale production methods must ensure that the exosomes retain their functional properties without contamination. However, practical challenges such as standardizing exosome isolation, targeted delivery, and stability remain significant.

Combining hydrogels with exosomes yields advanced biomaterials that can modify the wound inflammatory microenvironment, boosting vascularization, improving re-epithelialization, and accelerating wound healing by improving their stability and sustained release [129,130,131,132]. Yang et al. have successfully constructed a scaffold using a composite of thermosensitive pluronic F-127 and hUCMSC-exos for treating diabetic wounds. This hydrogel can facilitate the sustained release of exosomes to augment angiogenesis at the wound site. Such treatment accelerated wound re-epithelialization, improved regeneration, and showed a better quality of healing and skin remodeling [129]. Zhou et al. also reported a scaffold of pluronic F-127 with hADSCs-Exos that promoted re-epithelialization, facilitated collagen synthesis, up-regulated expression of skin barrier proteins, and reduced inflammation [133]. Hao et al. isolated EVs from PMSCs and, using integrin-based binding technology, immobilized these EVs onto an ECM-mimicking scaffold. The modified scaffold showed pro-angiogenic capacity and prevented EC apoptosis in an ischemic environment [134]. Therefore, the porous nature of scaffolds allows high surface area, more fluid and gas exchange and promotes cell adhesion, proliferation, migration, and differentiation for urging skin regeneration [89,128].

## 5. Bioengineered Exosomes Are Emerging as a Novel Tool for Chronic Wound Therapy

One key element in adult tissue that remains understudied is the role of numerous cell types, such as the resident fibroblasts, keratinocytes, endothelial cells, platelets, and blood-borne myeloid cells, at the wound site [135,136,137]. Exosomes from these cell types serve as intercellular messengers, orchestrating multidirectional communication between the resident cells and the blood-borne immune cells [138,139,140,141]. Under hyperglycemic conditions, keratinocyte-derived exosomes, overexpressing metastasis-associated lung adenocarcinoma transcript 1 (MALAT1), have been shown to improve macrophage functions such as phagocytic capacity, reparative phenotypic polarization, and reduced macrophage apoptotic activity. MALAT1 acts as a competitive antagonist of miR-1914-3p, accelerating milk fat globule epidermal growth factor 8 (MFGE8) production, affecting macrophage activities and TGF-β1/suppressor of mothers against decapentaplegic 3 (SMAD3) signaling cascade. By lowering apoptosis rates and promoting a pro-regenerative phenotype, macrophage-mediated phagocytosis enhances skin wound healing [142,143]. Additionally, keratinocyte-derived exosomes maintain the activity of macrophage and its migration by upregulating anti-inflammatory molecules and reducing the inflammatory mediators expressions, such as TNF-α, cluster of differentiation 74 (CD74), and inducible nitric oxide synthase (iNOS) [78,89,144]. Concurrently, macrophage-derived exosomes increase the gene transcriptional activity of vascular endothelial growth factor (VEGF), a crucial protein involved in angiogenesis and tissue regeneration [89]. These exosomes transition pro-inflammatory macrophages to a pro-regenerative phenotype, creating a milieu conducive to effective diabetic wound healing while also lowering levels of pro-inflammatory cytokines like TNF-α and interleukin-6 (IL-6) [145]. Dermal fibroblast exosomes (DF-Ex) have demonstrated the ability to stimulate tubule formation, migration, and proliferation of endothelial cells in umbilical vein in vitro, under normal and high glucose conditions, validating that DF-Ex has angiogenic potential [146]. Dermal fibroblasts exosomes subcutaneously injected into diabetic rats enhances wound healing by accelerating angiogenesis, collagen deposition, and remodeling of epithelial, and mitigating inflammation [147]. Han et al. [146] and Hu et al. [148] have shown that exosomes derived from three-dimensional spheroids (HDF XOs) significantly reduce matrix metalloproteinase-1 (MMP-1) expression and primarily enhance collagen type I expression by downregulating TNF-α and upregulating TGF-β, suggesting their potential role in chronic wound healing. Additionally, exosomes derived from hypoxic endothelial cells have been demonstrated to stimulate angiogenesis at the wound edge and improve oxygen concentration levels, thereby accelerating wound healing [146,148].

One major challenge in chronic wound healing is the high phagocytic potential of pro-inflammatory macrophages, which dominate chronic wound environments. This high phagocytic activity means that exosomes often fail to reach the targeted cells. Qie et al. demonstrated that traditionally activated macrophages (pro-inflammatory) have a greater phagocytic capacity than alternatively activated macrophages (anti-inflammatory), with stimulated macrophages exhibiting the highest phagocytic activity [89,149]. Thus, the engineered exosomes must be designed to evade the heightened phagocytic response of pro-inflammatory macrophages. This can be achieved through surface modifications and encapsulation techniques like fusion with proteins or liposomes [150], genetic engineering, covalent modification, non-covalent modification like ligand–receptor interaction, hydrophobic interaction, aptamer-based modification, and radioactive isotope labeling techniques of exosomes for imaging purposes to enhance the stability and targeting capabilities of exosomes. Matsumoto et al. used a genetic engineering approach for the synthesis of 125I-SAV-LA-Exos [151]. Wang et al. synthesized biotin-attached human umbilical vein endothelial cell (HUVEC)-derived exosomes through avidin–biotin interaction [152]. Addressing the rapid clearance and high phagocytic uptake of exosomes through advanced engineering and delivery strategies will enhance their therapeutic potential in chronic wound healing, enabling sustained bioactivity and targeted delivery to promote efficient tissue repair and healing processes [153,154]. An ideal molecularly targeted wound healing dressing should possess functionalities like tissue adhesiveness, controlled biodegradability, hemostatic efficacy, antimicrobial qualities, ultraviolet (UV) protection, self-healing abilities, appropriate mechanical properties, evading MPS, and a slow release of bioactive molecules to effectively support the healing process [155].

**Figure 1:**
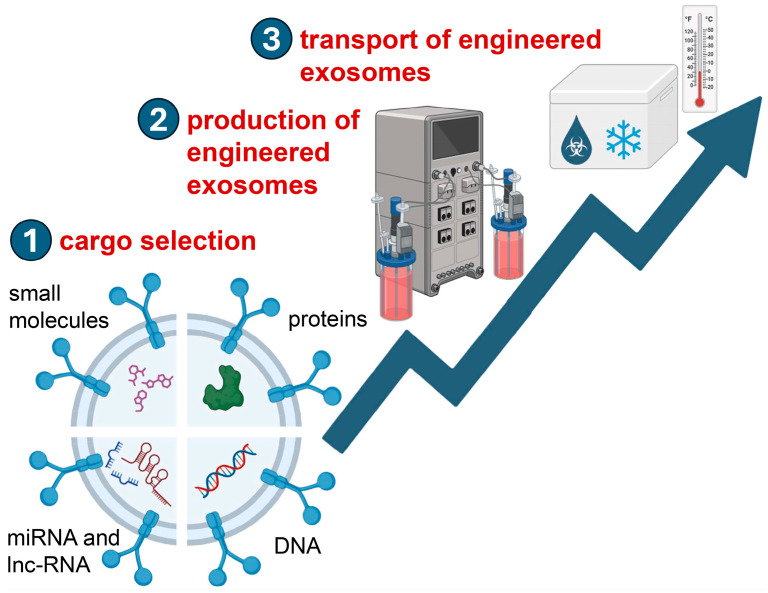
The three most critical steps in the therapeutic applications of engineered exosomes. First, the selection of appropriate cargo(s); second, the production process; and third, the storage and transport of the therapeutic-engineered exosomes. Figure created with Biorender.com.

Recognized as a leading delivery system in biomedicine, exosomes offer several advantages: reduced toxicity, little immunogenic potential, nanoscale size allowing deeper tissue penetration, flexibility in carrying molecular payload, and surface engineering capabilities [156,157,158,159]. Compared to conventional nanoparticles [160], exosomes exhibit improved stability in circulation, reduced immunogenicity, enhanced biocompatibility, and tissue-specific homing efficiency, making them a promising alternative to cell-based therapy [161,162,163]. The therapeutic use of exosomes is still in its early phases, and issues like exosome wastage and the need for repeated administration at wound sites raise costs and lower treatment compliance (Figure 1).

Furthermore, nanoengineering approaches have been employed to create exosome-derived biomimetic vesicles [98,164,165]. Exosome engineering techniques like genetic engineering, surface modification, exosome cargo loading, fusion protein targeting, incubation with membrane permeabilizers, and physical engineering [166]. Exosome surface modification can be achieved by using a crosslinking reaction such as click chemistry, and it shows no alterations in exosome size and function [167]. Liang et al. have developed a hybrid membrane strategy by the fusion of exosomes with liposomes containing polyethylene glycol (PEG) to deliver the CRISPR-Cas9 system for targeted gene editing [168]. Gene modification techniques can be utilized to modify specific sites and improve the functionality of exosomes [169]. Alvarez Erviti et al. have genetically modified Dendritic cells (DCs) to express fusion proteins and loaded the bioengineered exosomes with siRNA targeting the central nervous system [150]. The bioengineering of exosomes not only increases the exosome delivery but also minimizes the out-of-target side effects [170]. These engineered exosomes maintain the structural integrity of the exosome membrane [170]. This enhances the therapeutic benefits of exosomes and addresses the technological issues that currently limit their clinical application [171]. Some already available drug delivery systems are hybrid nanoparticles, lipid-based emulsions, oral in situ gel delivery systems, micro electro mechanical systems (MEMS), etc., which also show no target specificity, poor absorption from the administration site, premature secretion from the body, premature metabolism of the drug, poor bioavailability, repeated dosing, the toxicity of materials used, higher manufacturing cost, and poor patient compliance. The development of delivery platforms in the form of bioengineered exosomes is a critical component to attract significant research attention. Antes et al. modified glycero-phospholipid-PEG with vesicular lipid bilayer membranes to construct an exosome membrane platform, where biotinylated molecules can be coupled for vesicle decoration, and it showed improved uptake and on-site target [172]. These exosome-based drug delivery platforms with modified surfaces show the improved stability, efficient delivery, accuracy, decreased immunogenicity [173], and therapeutic potential of exosome-mediated cargo delivery in drug delivery systems and regenerative medicine [173].

The number of clinical studies in chronic wound healing using exosomes or extracellular vesicles are limited and their therapeutic effects need to be explored. Upon searching keywords like chronic wounds, exosomes, and extracellular vesicles in clinicaltrials.gov, only a limited number of studies are present. Clinical study number NCT04761562 assesses the effectiveness of platelet- and extracellular vesicle-rich plasma, an autologous blood-derived product, as a supplement to surgical therapy of chronic tympanic membrane perforations. Another study, NCT04134676, investigated the therapeutic potential of Conditioned Medium Stem Cells as an additional growth factor in chronic skin ulcer healing and compared the outcome of chronic ulcer healing in patients receiving this treatment. In study number NCT04928534, high-throughput screening and multi-omics (transcriptomics and proteomics) combined analytic technologies were employed to investigate possible chronic traumatic encephalopathy (CTE)/traumatic encephalopathy syndrome (TES) biomarkers (RNA and protein) in blood and exosomes. Thereafter, these biomarkers were then integrated with the previously published traumatic brain injury (TBI) biomarkers to create a new set of CTE/TES molecular diagnostic signatures. The discoveries may pave the way for a new clinical diagnosis of the condition as well as future study into its therapy strategy.

**Figure 2:**
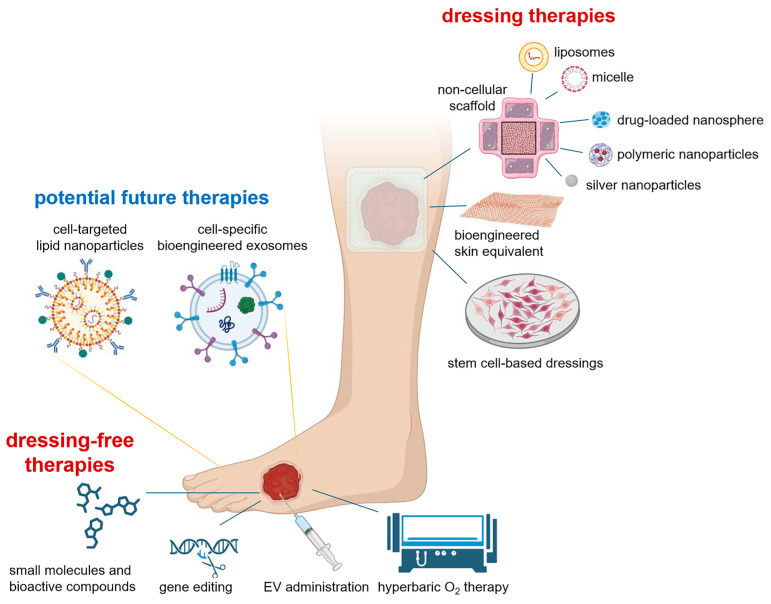
Chronic wound therapies can be of two types. First, one that involves dressing such as stem cell-based dressings, bioengineered skin equivalent, and non-cellular scaffolds that can incorporate active biomolecules loaded in liposomes, micelles, nanospheres, polymeric nanoparticles, and inorganic nanoparticles such as silver. Second, several dressing-free therapies involve small molecules and bioactive compounds, gene editing, direct EV administration, or hyperbaric oxygen therapy. Between these two modalities, the potential therapeutics can be cell-specific bioengineered exosomes or/and cell-targeted lipid nanoparticles. Figure created with Biorender.com.

To advance exosome engineering and exosome-mediated delivery systems from theoretical concepts to practical and existing clinical applications, comprehensive and interdisciplinary research efforts are urgently needed (Figure 2). These efforts should focus on improving the therapeutic outcomes of exosome-based biomimetic drug delivery systems in chronic wounds. Such research must integrate expertise from fields such as nanotechnology, materials science, molecular biology, and clinical medicine to develop innovative solutions that address the current limitations and enhance the efficacy of exosome-based therapies.

## 6. Targeted Exosome Delivery

To enable targeted delivery of exosomes to specific cell types, transport routes, and internal cell compartments, as well as to have more control over overdosing, biodistribution, and therapeutic action, rational exosome engineering strategies can be employed (Table 2) [168]. These strategies involve manipulating the morphological and surface physicochemical features of exosomes such that they interact predictably with physiological components such as proteins and cells [157,174]. For the development of efficient exosome-based delivery platforms in chronic wound healing, several strategies are proposed to enhance therapeutic outcomes:

1. Understanding endocytosis: The endocytosis of exosomes is critical for maximizing exosome uptake and cellular targeting. The endocytic pathways vary depending on the cell type and the source of exosomes. For example, although Joshi et al. has shown that the uptake of vesicles by cells involves endocytosis [175], it has been reported that clathrin-mediated endocytosis and micropinocytosis are predominant process for cellular uptake of PC12 cell-derived exosomes. Decoding the specific endocytic mechanisms will enable the design of exosomes that are more effectively internalized by target cells [153,176,177].
2. Preventing MPS internalization: When exosomes encounter physiological fluids like blood or lymph, they can interact with biomolecules such as opsonins, facilitating cellular detection and clearance by the MPS. To improve exosome-based targeted delivery and prevent MPS internalization, the concept of host “bioinvisibility” is crucial. One strategy involves coating the surface of exosomes with self-identifying proteins, such as CD47, which binds to the SIRP-alpha receptor and helps evade the immune system, thereby prolonging circulation time. Recent studies have shown that attaching the active binding sequence of CD47 to exosome surfaces decreases MPS absorption and significantly lengthens circulation durations [178].

**Table 2:** The potential use of exosomes derived from different cell types in wound healing.

Cell Source	Function	Year	References
Keratinocytes	Enhance macrophage functions by overexpressing MALAT1	2023	[179]
	Accelerate migration and proliferation of keratinocytes and fibroblasts via MAPK pathways	2021	[180]
	Modulate number and function of macrophages	2020	[141]
	Alter VEGF and fibroblast growth factors (FGF) and activate fibroblasts and endothelial cell migration	2020	[181]
Macrophages	Promote osteogenesis through microRNA-21a-5p	2022	[182]
	Increase VEGF expression causing proliferation and migration of endothelial cells	2019	[145]
	Increase expression of VEGF, Wnt3a, and miR-130a to promote angiogenesis, fibroblast proliferation, and re-epithelialization	2020	[183]
	Promote angiogenesis, proliferation, granulation tissue formation, and collagen accumulation by overexpressing miR-223	2022	[184]
	Promote wound closure and re-epithelialization by switching the expression of iNOS to arginase	2022	[185]
Fibroblasts	Upregulates the expression of collagen type I and TGFβ	2019	[148]
	Promote re-epithelialization, proliferation, and inhibit inflammation via β-catenin signaling pathway	2021	[146]
	Transition of fibroblasts to myofibroblasts	2022	[147]
	Promote fibroblast migration and transformation	2022	[147]

3. Using host “self” identification signals: Employing host “self” identification signals to lessen complement activation and phagocytic recognition is a promising approach. For example, Factor H, a cofactor of Factor I, deactivates the complement pathway by promoting the dissociation of the Bb complex and cleavage of C3b. Researchers employed sialic acid, a component found on the pathogen surface, to bind Factor H and avoid complement activation and immune detection [186].
4. Surface energy modifications: Modifying the surface energies of exosomes, such as hydrophilicity/hydrophobicity, can reduce protein adsorption and phagocytic recognition. Hydrophilic poly (ethylene glycol) (PEG) is often immobilized to create a steric barrier, decreasing protein adsorption and extending blood circulation times for nanoparticles [187]. Qie et al. demonstrated that adding PEG to nanoparticles reduces clearance by all macrophage phenotypes while coating nanoparticles with CD47 specifically reduces phagocytic activity by pro-inflammatory macrophages [149,188].
5. Developing immune-tolerant nanomedicines: Surface modifications of exosomes that enable immune system evasion to offer a rational method for creating immune-tolerant nanomedicines. Further research is needed to develop safe, secure, and efficient methods to deactivate the MPS. One potential goal is to create a highly effective and universal blocker that can avoid dose-related toxicity associated with traditional MPS blocking methods [189].

However, adding more functionalities to exosomes increases their complexity, making them harder to scale and reproduce, which could impede their application in clinical settings. Therefore, while enhancing exosome functionality is critical, it must be balanced with considerations for manufacturability and clinical translation [154].

## 7. Balancing Boundaries: Navigating Stringency and Innovation in Chronic Wound Healing

The role of the regulatory bodies in chronic wound healing therapeutics is crucial, yet it is not without its challenges and criticisms. While these boards are instrumental in ensuring ethical standards and regulatory compliance, several factors can hamper their effectiveness [190]. Such bodies have stringent guidelines to conduct the research ethically and safely. However, the rigidity of these guidelines sometimes stifles innovation, whereas new therapies require flexible and adaptive regulatory approaches. The review process involves scrutiny of protocols and informed consent forms, ensuring that methodologies are scientifically sound and ethically justified. The complexity and length of consent forms can sometimes overwhelm patients, especially those with chronic conditions who may already be dealing with significant stress and discomfort. While this thoroughness is commendable, this bureaucratic nature can also lead to significant delays in the approval process that can be detrimental to time-sensitive research.

The transparency related to the awareness of the participants regarding the nature of the study, dangers, advantages, and rights is vital. Once a study is approved, continuous oversight through regular monitoring of progress reports, adverse event reports, and protocol amendments is essential for maintaining participant safety. Different regulatory bodies have different standards and interpretations, leading to inconsistencies in the review process. This can be challenging for multi-site studies, where approval from multiple regulatory bodies is needed. Also, the conflicts of interest between members and researchers sometimes can compromise the objectivity of the review process. However, resource constraints, including limited time, staff, and funding, can impede their ability to provide effective ongoing oversight. This can result in delayed responses to emerging risks and insufficient follow-up on reported issues. The bodies strive to stay informed about the latest scientific advancements, but the rapidly evolving nature of these fields can outpace their ability to keep up to date. This can sometimes hinder the progress of novel therapies that could offer significant benefits to patients.

## 8. Conclusions and Future Perspectives

The development of novel tools for improving wound healing is a promising yet challenging endeavor, particularly for chronic wounds. Significant progress has been made in understanding the pathophysiological processes driving injury, which is crucial for developing targeted therapeutic strategies. Transdisciplinary collaborations are essential to define unmet clinical needs and reshape research goals to align with Food and Drug Administration (FDA) guidelines. Transitioning from unspecific to molecularly targeted wound dressings require the early identification of these needs through collaboration and targeted customer discovery. Further research should focus on several key areas to overcome existing challenges and enhance therapeutic efficacy in chronic wound healing. The emphasis on molecular targeting using exosome-based therapies can address specific pathophysiologic factors and help in exploring personalized treatment approaches based on individual patient profiles and wound characteristics. The hydrogel-based approach, 3D bioprinting, and platelet-rich plasma-based treatments can be used for the successful delivery of exosome-based systems and to minimize the adverse effects. The therapies must focus on mimicking the skin’s extracellular matrix environment to enhance skin regeneration. Before that, the challenges included isolation of pure populations of exosomes, as till today, researchers are using a combined population of extracellular vesicles instead of exosomes. Hence, its purification, production, standardization, scalable production, contamination levels, and safety regarding their use must be addressed beforehand. The synthetic approaches used are very complex and are not able to isolate specific subpopulations. Another major hurdle while working with exosomes is that the exosomes have poor retention at the wound surface, hence, modified systems are needed for slow release for maximum efficacy. The safety of treatment using exosome-based therapies also depends upon the quality of exosomes utilized, how they were administered, and the health of the patient (including allergic reactions, infection, or inflammation at the injection site). There is also a reduced risk of immune rejection as these can be derived from a person’s cells. Currently, the exosome for clinical use is very limited due to low yield and high cost. The incorporation of advanced technologies will drive the development of safe and effective therapies, in future benefiting patients with chronic wound conditions.

## Figures and Tables

**Table 1:** Nanotherapeutics employed in different stages of wound healing.

Types of Nanomaterials	Biomolecules Loaded	Role in Wound Healing	References
Polymeric nanoparticles	Drugs, nitric oxide, curcumin, siRNA	Hemostasis, proliferation, inflammation, remodeling	[101]
Zinc Oxide nanoparticles		Hemostasis	[102]
Nanoceria		Hemostasis, inflammation, remodeling	[103]
Gold nanoparticles	Drugs, siRNA	Proliferation, inflammation	[104]
Fullerene, Graphene Oxide, Carbon nanotubes		Proliferation, inflammation	[105,106]
Zinc Oxide nanoflowers		Proliferation	[107]
Polymeric nanofibers	Plasmid DNA	Proliferation	[108]
Polymeric nanoscaffolds	Stem cells	Proliferation, remodeling	[109]
Bioactive glass particles		Proliferation	[110]
Dendrimers	Plasmid DNA	Proliferation	[111]
Liposomes	Growth factor, drugs	Proliferation, inflammation	[112]
Copper nanoparticles		Inflammation	[113]
Silver nanoparticles	Drugs, oligonucleotide	Inflammation	[114]
Ceramic nanoparticles	Nitric oxide, curcumin	Inflammation	[103]
Iron Oxide nanoparticles	Nitric oxide	Remodeling	[115]
Metal–Organic Frameworks (M- Zn, Cu, Fe, Mg, Ag, and others)	Drugs	Hemostasis, proliferation, inflammation, remodeling	[116,117]

## Abbreviations

HDF XOs	exosomes derived from three-dimensional spheroids
3D	three-dimensional
CD47	cluster of differentiation 47
CD74	cluster of differentiation 74
CD206	cluster of differentiation 206
CTE	chronic traumatic encephalopathy
DC	dendritic cells
DF-Ex	dermal fibroblasts exosomes
DNA	deoxyribonucleic acid
ECM	extracellular matrix
sEVs	small extracellular vesicles
FDA	food and drug administration
FGF	fibroblast growth factors
HUVEC	human umbilical vein endothelial cell
IL-6	interleukin-6
IL-1β	interleukin-1 beta
iNOS	inducible nitric oxide synthase
LPS	lipopolysaccharides
MALAT1	metastasis-associated lung adenocarcinoma transcript 1
MEMS	micro electro mechanical systems
MFGE8	milk fat globule epidermal growth factor 8
miRNA	micro ribonucleic acid
MMP-1	matrix metalloproteinase-1
MPS	mononuclear phagocyte system
MOFs	metal-organic frameworks
MRSA	methicillin-resistant *Staphylococcus aureus*
MSC	mesenchymal stem cell
NPWT	negative pressure wound therapy
PEG	poly(ethylene glycol)
ROS	reactive oxygen species
SMAD3	suppressor of mothers against decapentaplegic 3
TBI	traumatic brain injury
TES	traumatic encephalopathy syndrome
TGF-β	transforming growth factor-beta
TGFβR	transforming growth factor beta receptor
TIME	tissue, inflammation/infection, moisture, edge
TNF-α	tumor necrosis factor-alpha
TWA	triangle of wound assessment
UV	ultraviolet
VEGF	vascular endothelial growth factor
Wnt3a	wingless-type MMTV integration site family, member 3A

## References

[1] Frykberg R.G., Banks J. (2015). Challenges in the Treatment of Chronic Wounds. Adv. Wound Care.

[2] Eriksson E., Liu P.Y., Schultz G.S., Martins-Green M.M., Tanaka R., Weir D., Gould L.J., Armstrong D.G., Gibbons G.W., Wolcott R. (2022). Chronic wounds: Treatment consensus. Wound Repair. Regen..

[3] Gnyawali S.C., Sinha M., El Masry M.S., Wulff B., Ghatak S., Soto-Gonzalez F., Wilgus T.A., Roy S., Sen C.K. (2020). High resolution ultrasound imaging for repeated measure of wound tissue morphometry, biomechanics and hemodynamics under fetal, adult and diabetic conditions. PLoS ONE.

[4] Gnyawali S.C., Blum K., Pal D., Ghatak S., Khanna S., Raoy S., Sen C.K. (2017). Retooling Laser Speckle Contrast Analysis Algorithm to Enhance Non-Invasive High Resolution Laser Speckle Functional Imaging of Cutaneous Microcirculation. Sci. Rep..

[5] Mills S.E.E., Nicolson K.P., Smith B.H. (2019). Chronic pain: A review of its epidemiology and associated factors in population-based studies. Br. J. Anaesth..

[6] Klein T.M., Andrees V., Kirsten N., Protz K., Augustin M., Blome C. (2021). Social participation of people with chronic wounds: A systematic review. Int. Wound J..

[7] Sen C.K. (2021). Human Wound and Its Burden: Updated 2020 Compendium of Estimates. Adv. Wound Care.

[8] Sen C.K. (2019). Human Wounds and Its Burden: An Updated Compendium of Estimates. Adv. Wound Care.

[9] Monika P., Chandraprabha M.N., Rangarajan A., Waiker P.V., Chidambara Murthy K.N. (2021). Challenges in Healing Wound: Role of Complementary and Alternative Medicine. Front. Nutr..

[10] Sharma A., Shankar R., Yadav A., Pratap A., Ansari M., Srivastava V. (2024). Burden of Chronic Nonhealing Wounds: An Overview of the Worldwide Humanistic and Economic Burden to the Healthcare System. Int. J. Low. Extrem. Wounds.

[11] Ffrench C., Finn D., Velligna A., Ivory J., Healy C., Butler K., Sezgin D., Carr P., Probst S., McLoughlin A. (2023). Systematic review of topical interventions for the management of pain in chronic wounds. Pain Rep..

[12] Järbrink K., Ni G., Sönnergren H., Schmidtchen A., Pang C., Bajpai R., Car J. (2017). The humanistic and economic burden of chronic wounds: A protocol for a systematic review. Syst. Rev..

[13] Hoversten K.P., Kiemele L.J., Stolp A.M., Takahashi P.Y., Verdoorn B.P. (2020). Prevention, Diagnosis, and Management of Chronic Wounds in Older Adults. Mayo Clin. Proc..

[14] Sen C., Roy S., Gordillo G., Neligan P.C. (2017). Wound Healing. Plastic Surgery: Volume One.

[15] Gallego-Perez D., Pal D., Ghatak S., Malkoc V., Higuita-Castro N., Gnyawali S., Chang L., Liao W.C., Shi J., Sinha M. (2017). Topical tissue nano-transfection mediates non-viral stroma reprogramming and rescue. Nat. Nanotechnol..

[16] Seth I., Lim B., Cevik J., Gracias D., Chua M., Kenney P.S., Rozen W.M., Cuomo R. (2024). Impact of nutrition on skin wound healing and aesthetic outcomes: A comprehensive narrative review. JPRAS Open.

[17] Hicks C.W., Selvarajah S., Mathioudakis N., Sherman R.E., Hines K.F., Black J.H., Abularrage C.J. (2016). Burden of Infected Diabetic Foot Ulcers on Hospital Admissions and Costs. Ann. Vasc. Surg..

[18] Namgoong S., Baik S., Han S.K., Son J.W., Kim J.Y. (2023). Developing and Establishing a Wound Dressing Team: Experience and Recommendations. J. Korean Med. Sci..

[19] Kolimi P., Narala S., Nyavanandi D., Youssef A.A.A., Dudhipala N. (2022). Innovative Treatment Strategies to Accelerate Wound Healing: Trajectory and Recent Advancements. Cells.

[20] Durant F., Whited J.L. (2021). Finding Solutions for Fibrosis: Understanding the Innate Mechanisms Used by Super-Regenerator Vertebrates to Combat Scarring. Adv. Sci..

[21] Karppinen S.M., Heljasvaara R., Gullberg D., Tasanen K., Pihlajaniemi T. (2019). Toward understanding scarless skin wound healing and pathological scarring. F1000Research.

[22] Han G., Ceilley R. (2017). Chronic Wound Healing: A Review of Current Management and Treatments. Adv. Ther..

[23] Holzinger D., Gieldon L., Mysore V., Nippe N., Taxman D.J., Duncan J.A., Broglie P.M., Marketon K., Austermann J., Vogl T. (2012). Staphylococcus aureus Panton-Valentine leukocidin induces an inflammatory response in human phagocytes via the NLRP3 inflammasome. J. Leukoc. Biol..

[24] Li M., Hou Q., Zhong L., Zhao Y., Fu X. (2021). Macrophage Related Chronic Inflammation in Non-Healing Wounds. Front. Immunol..

[25] Nirenjen S., Narayanan J., Tamilanban T., Subramaniyan V., Chitra V., Fuloria N.K., Wong L.S., Ramachawolran G., Sekar M., Gupta G. (2023). Exploring the contribution of pro-inflammatory cytokines to impaired wound healing in diabetes. Front. Immunol..

[26] Bluestein D., Javaheri A. (2008). Pressure ulcers: Prevention, evaluation, and management. Am. Fam. Physician.

[27] Mieczkowski M., Mrozikiewicz-Rakowska B., Kowara M., Kleibert M., Czupryniak L. (2022). The Problem of Wound Healing in Diabetes-From Molecular Pathways to the Design of an Animal Model. Int. J. Mol. Sci..

[28] Yachmaneni A., Jajoo S., Mahakalkar C., Kshirsagar S., Dhole S. (2023). A Comprehensive Review of the Vascular Consequences of Diabetes in the Lower Extremities: Current Approaches to Management and Evaluation of Clinical Outcomes. Cureus.

[29] Burgess J.L., Wyant W.A., Abdo Abujamra B., Kirsner R.S., Jozic I. (2021). Diabetic Wound-Healing Science. Medicina.

[30] Bootun R. (2013). Effects of immunosuppressive therapy on wound healing. Int. Wound J..

[31] Ridiandries A., Tan J.T.M., Bursill C.A. (2018). The Role of Chemokines in Wound Healing. Int. J. Mol. Sci..

[32] Jacobson L.K., Johnson M.B., Dedhia R.D., Niknam-Bienia S., Wong A.K. (2017). Impaired wound healing after radiation therapy: A systematic review of pathogenesis and treatment. JPRAS Open.

[33] Huang R.-X., Zhou P.-K. (2020). DNA damage response signaling pathways and targets for radiotherapy sensitization in cancer. Signal Transduct. Target. Ther..

[34] Bouten R.M., Young E.F., Selwyn R., Iacono D., Rittase W.B., Day R.M., Gorbunov N.V. (2021). Chapter Two—Effects of radiation on endothelial barrier and vascular integrity. Tissue Barriers in Disease, Injury and Regeneration.

[35] McCarty S.M., Percival S.L. (2013). Proteases and Delayed Wound Healing. Adv. Wound Care.

[36] Sen C.K., Ghatak S., Gnyawali S.C., Roy S., Gordillo G.M. (2016). Cutaneous Imaging Technologies in Acute Burn and Chronic Wound Care. Plast. Reconstr. Surg..

[37] Stojadinovic O., Pastar I., Vukelic S., Mahoney M.G., Brennan D., Krzyzanowska A., Golinko M., Brem H., Tomic-Canic M. (2008). Deregulation of keratinocyte differentiation and activation: A hallmark of venous ulcers. J. Cell Mol. Med..

[38] Nunan R., Harding K.G., Martin P. (2014). Clinical challenges of chronic wounds: Searching for an optimal animal model to recapitulate their complexity. Dis. Models Mech..

[39] Tsou Y.H., Khoneisser J., Huang P.C., Xu X. (2016). Hydrogel as a bioactive material to regulate stem cell fate. Bioact. Mater..

[40] Powers J.G., Higham C., Broussard K., Phillips T.J. (2016). Wound healing and treating wounds: Chronic wound care and management. J. Am. Acad. Dermatol..

[41] Diban F., Di Lodovico S., Di Fermo P., D’Ercole S., D’Arcangelo S., Di Giulio M., Cellini L. (2023). Biofilms in Chronic Wound Infections: Innovative Antimicrobial Approaches Using the In Vitro Lubbock Chronic Wound Biofilm Model. Int. J. Mol. Sci..

[42] Roy S., Elgharably H., Sinha M., Ganesh K., Chaney S., Mann E., Miller C., Khanna S., Bergdall V.K., Powell H.M. (2014). Mixed-species biofilm compromises wound healing by disrupting epidermal barrier function. J. Pathol..

[43] Roy S., Santra S., Das A., Dixith S., Sinha M., Ghatak S., Ghosh N., Banerjee P., Khanna S., Mathew-Steiner S. (2020). Staphylococcus aureus Biofilm Infection Compromises Wound Healing by Causing Deficiencies in Granulation Tissue Collagen. Ann. Surg..

[44] Haesler E., Swanson T., Ousey K., Carville K. (2019). Clinical indicators of wound infection and biofilm: Reaching international consensus. J. Wound Care.

[45] Wu Y.K., Cheng N.C., Cheng C.M. (2019). Biofilms in Chronic Wounds: Pathogenesis and Diagnosis. Trends Biotechnol..

[46] Ganesh K., Sinha M., Mathew-Steiner S.S., Das A., Roy S., Sen C.K. (2015). Chronic Wound Biofilm Model. Adv. Wound Care.

[47] Sen C.K., Ghatak S. (2015). miRNA control of tissue repair and regeneration. Am. J. Pathol..

[48] Ghatak S., Chan Y.C., Khanna S., Banerjee J., Weist J., Roy S., Sen C.K. (2015). Barrier Function of the Repaired Skin Is Disrupted Following Arrest of Dicer in Keratinocytes. Mol. Ther..

[49] Wang X., Yuan C.X., Xu B., Yu Z. (2022). Diabetic foot ulcers: Classification, risk factors and management. World J. Diabetes.

[50] Ghatak S., Hemann C., Boslett J., Singh K., Sharma A., El Masry M.S., Abouhashem A.S., Ghosh N., Mathew-Steiner S.S., Roy S. (2023). Bacterial Pyocyanin Inducible Keratin 6A Accelerates Closure of Epithelial Defect under Conditions of Mitochondrial Dysfunction. J. Investig. Dermatol..

[51] Singh K., Rustagi Y., Abouhashem A.S., Tabasum S., Verma P., Hernandez E., Pal D., Khona D.K., Mohanty S.K., Kumar M. (2022). Genome-wide DNA hypermethylation opposes healing in patients with chronic wounds by impairing epithelial-mesenchymal transition. J. Clin. Investig..

[52] Schilrreff P., Alexiev U. (2022). Chronic Inflammation in Non-Healing Skin Wounds and Promising Natural Bioactive Compounds Treatment. Int. J. Mol. Sci..

[53] Das A., El Masry M.S., Gnyawali S.C., Ghatak S., Singh K., Stewart R., Lewis M., Saha A., Gordillo G., Khanna S. (2019). Skin Transcriptome of Middle-Aged Women Supplemented with Natural Herbo-mineral Shilajit Shows Induction of Microvascular and Extracellular Matrix Mechanisms. J. Am. Coll. Nutr..

[54] Zeng R., Lin C., Lin Z., Chen H., Lu W., Lin C., Li H. (2018). Approaches to cutaneous wound healing: Basics and future directions. Cell Tissue Res..

[55] Weigelt M.A., Lev-Tov H.A., Tomic-Canic M., Lee W.D., Williams R., Strasfeld D., Kirsner R.S., Herman I.M. (2022). Advanced Wound Diagnostics: Toward Transforming Wound Care into Precision Medicine. Adv. Wound Care.

[56] Gil S.B. (2020). Implementing the Triangle of Wound Assessment framework to transform the care pathway for diabetic foot ulcers. J. Wound Care.

[57] Gushiken L.F.S., Beserra F.P., Bastos J.K., Jackson C.J., Pellizzon C.H. (2021). Cutaneous Wound Healing: An Update from Physiopathology to Current Therapies. Life.

[58] Hess C.T. (2019). Comprehensive Patient and Wound Assessments. Adv. Ski. Wound Care.

[59] Roy S., Sen C.K., Ghatak S., Higuita-Castro N., Palakurti R., Nalluri N., Clark A., Stewart R., Gallego-Perez D., Prater D.N. (2020). Neurogenic tissue nanotransfection in the management of cutaneous diabetic polyneuropathy. Nanomedicine.

[60] Freedman B.R., Hwang C., Talbot S., Hibler B., Matoori S., Mooney D.J. (2023). Breakthrough treatments for accelerated wound healing. Sci. Adv..

[61] Queen D., Harding K. (2023). What’s the true costs of wounds faced by different healthcare systems around the world?. Int. Wound J..

[62] Sen C.K., Roy S. (2019). Sociogenomic Approach to Wound Care: A New Patient-Centered Paradigm. Adv. Wound Care.

[63] Espaulella-Ferrer M., Espaulella-Panicot J., Noell-Boix R., Casals-Zorita M., Ferrer-Sola M., Puigoriol-Juvanteny E., Cullell-Dalmau M., Otero-Viñas M. (2021). Assessment of frailty in elderly patients attending a multidisciplinary wound care centre: A cohort study. BMC Geriatr..

[64] Williams J., Allen L., Wickramasinghe K., Mikkelsen B., Roberts N., Townsend N. (2018). A systematic review of associations between non-communicable diseases and socioeconomic status within low- and lower-middle-income countries. J. Glob. Health.

[65] Cole S.W., Conti G., Arevalo J.M., Ruggiero A.M., Heckman J.J., Suomi S.J. (2012). Transcriptional modulation of the developing immune system by early life social adversity. Proc. Natl. Acad. Sci. USA.

[66] Idaghdour Y., Czika W., Shianna K.V., Lee S.H., Visscher P.M., Martin H.C., Miclaus K., Jadallah S.J., Goldstein D.B., Wolfinger R.D. (2010). Geographical genomics of human leukocyte gene expression variation in southern Morocco. Nat. Genet..

[67] Zhao A., Sun J., Liu Y. (2023). Understanding bacterial biofilms: From definition to treatment strategies. Front. Cell Infect. Microbiol..

[68] Tian F., Li J., Nazir A., Tong Y. (2021). Bacteriophage—A Promising Alternative Measure for Bacterial Biofilm Control. Infect. Drug Resist..

[69] Veličković V., Janković D. (2023). Challenges around quantifying uncertainty in a holistic approach to hard-to-heal wound management: Health economic perspective. Int. Wound J..

[70] Sen C.K. (2024). Standardized Wound Care: Patchwork Practices?. Adv. Wound Care.

[71] Sutherland B.L., Pecanac K., Bartels C.M., Brennan M.B. (2020). Expect delays: Poor connections between rural and urban health systems challenge multidisciplinary care for rural Americans with diabetic foot ulcers. J. Foot Ankle Res..

[72] Fayne R.A., Borda L.J., Egger A.N., Tomic-Canic M. (2020). The Potential Impact of Social Genomics on Wound Healing. Adv. Wound Care.

[73] Garima, Sharma D., Kumar A., Mostafavi E. (2023). Extracellular vesicle-based biovectors in chronic wound healing: Biogenesis and delivery approaches. Mol. Ther. Nucleic Acids.

[74] Nuutila K., Katayama S., Vuola J., Kankuri E. (2014). Human Wound-Healing Research: Issues and Perspectives for Studies Using Wide-Scale Analytic Platforms. Adv. Wound Care.

[75] Seaton M., Hocking A., Gibran N.S. (2015). Porcine models of cutaneous wound healing. ILAR J..

[76] Tottoli E.M., Dorati R., Genta I., Chiesa E., Pisani S., Conti B. (2020). Skin Wound Healing Process and New Emerging Technologies for Skin Wound Care and Regeneration. Pharmaceutics.

[77] Song Y., You Y., Xu X., Lu J., Huang X., Zhang J., Zhu L., Hu J., Wu X., Xu X. (2023). Adipose-Derived Mesenchymal Stem Cell-Derived Exosomes Biopotentiated Extracellular Matrix Hydrogels Accelerate Diabetic Wound Healing and Skin Regeneration. Adv. Sci..

[78] Joorabloo A., Liu T. (2022). Recent advances in nanomedicines for regulation of macrophages in wound healing. J. Nanobiotechnol..

[79] Almadani Y.H., Vorstenbosch J., Davison P.G., Murphy A.M. (2021). Wound Healing: A Comprehensive Review. Semin. Plast. Surg..

[80] Norman G., Shi C., Goh E.L., Murphy E.M., Reid A., Chiverton L., Stankiewicz M., Dumville J.C. (2022). Negative pressure wound therapy for surgical wounds healing by primary closure. Cochrane Database Syst. Rev..

[81] Oryan A., Alemzadeh E., Moshiri A. (2017). Burn wound healing: Present concepts, treatment strategies and future directions. J. Wound Care.

[82] Citeroni M.R., Ciardulli M.C., Russo V., Della Porta G., Mauro A., El Khatib M., Di Mattia M., Galesso D., Barbera C., Forsyth N.R. (2020). In Vitro Innovation of Tendon Tissue Engineering Strategies. Int. J. Mol. Sci..

[83] Orr S.B., Chainani A., Hippensteel K.J., Kishan A., Gilchrist C., Garrigues N.W., Ruch D.S., Guilak F., Little D. (2015). Aligned multilayered electrospun scaffolds for rotator cuff tendon tissue engineering. Acta Biomater..

[84] Ashley G.W., Henise J., Reid R., Santi D.V. (2013). Hydrogel drug delivery system with predictable and tunable drug release and degradation rates. Proc. Natl. Acad. Sci. USA.

[85] Lambers H., Piessens S., Bloem A., Pronk H., Finkel P. (2006). Natural skin surface pH is on average below 5, which is beneficial for its resident flora. Int. J. Cosmet. Sci..

[86] Kruse C.R., Singh M., Targosinski S., Sinha I., Sørensen J.A., Eriksson E., Nuutila K. (2017). The effect of pH on cell viability, cell migration, cell proliferation, wound closure, and wound reepithelialization: In vitro and in vivo study. Wound Repair. Regen..

[87] Sharma A., Mittal P., Yadav A., Mishra A.K., Hazari P.P., Sharma R.K. (2022). Sustained Activity of Stimuli-Responsive Curcumin and Acemannan Based Hydrogel Patches in Wound Healing. ACS Appl. Bio Mater..

[88] Liang Y., Zhao X., Hu T., Chen B., Yin Z., Ma P.X., Guo B. (2019). Adhesive Hemostatic Conducting Injectable Composite Hydrogels with Sustained Drug Release and Photothermal Antibacterial Activity to Promote Full-Thickness Skin Regeneration During Wound Healing. Small.

[89] Shi L., Song D., Meng C., Cheng Y., Wang B., Yang Z. (2024). Opportunities and challenges of engineered exosomes for diabetic wound healing. Giant.

[90] Zhong Y., Wei E.-t., Wu L., Wang Y., Lin Q., Wu N., Chen H., Tang N. (2024). Novel Biomaterials for Wound Healing and Tissue Regeneration. ACS Omega.

[91] Vach Agocsova S., Culenova M., Birova I., Omanikova L., Moncmanova B., Danisovic L., Ziaran S., Bakos D., Alexy P. (2023). Resorbable Biomaterials Used for 3D Scaffolds in Tissue Engineering: A Review. Materials.

[92] Nandhini J., Karthikeyan E., Rajeshkumar S. (2024). Nanomaterials for wound healing: Current status and futuristic frontier. Biomed. Technol..

[93] Afshar M., Rezaei A., Eghbali S., Nasirizadeh S., Alemzadeh E., Alemzadeh E., Shadi M., Sedighi M. (2024). Nanomaterial strategies in wound healing: A comprehensive review of nanoparticles, nanofibres and nanosheets. Int. Wound J..

[94] Kumar A., Sharma A., Chen Y., Jones M.M., Vanyo S.T., Li C., Visser M.B., Mahajan S.D., Sharma R.K., Swihart M.T. (2021). Copper@ZIF-8 Core-Shell Nanowires for Reusable Antimicrobial Face Masks. Adv. Funct. Mater..

[95] Marambio-Jones C., Hoek E.M.V. (2010). A review of the antibacterial effects of silver nanomaterials and potential implications for human health and the environment. J. Nanopart. Res..

[96] Keleştemur S., Kilic E., Uslu Ü., Cumbul A., Ugur M., Akman S., Culha M. (2012). Wound healing properties of modified silver nanoparticles and their distribution in mouse organs after topical application. Nano Biomed. Eng..

[97] Shaikh S., Nazam N., Rizvi S.M.D., Ahmad K., Baig M.H., Lee E.J., Choi I. (2019). Mechanistic insights into the antimicrobial actions of metallic nanoparticles and their implications for multidrug resistance. Int. J. Mol. Sci..

[98] Blanco-Fernandez B., Castaño O., Mateos-Timoneda M., Engel E., Pérez-Amodio S. (2021). Nanotechnology Approaches in Chronic Wound Healing. Adv. Wound Care.

[99] Xiao J., Zhu Y., Huddleston S., Li P., Xiao B., Farha O.K., Ameer G.A. (2018). Copper metal–organic framework nanoparticles stabilized with folic acid improve wound healing in diabetes. ACS Nano.

[100] Jiang Y., Han Y., Wang J., Lv F., Yi Z., Ke Q., Xu H. (2019). Space-oriented nanofibrous scaffold with silicon-doped amorphous calcium phosphate nanocoating for diabetic wound healing. ACS Appl. Bio Mater..

[101] Korrapati P.S., Karthikeyan K., Satish A., Krishnaswamy V.R., Venugopal J.R., Ramakrishna S. (2016). Recent advancements in nanotechnological strategies in selection, design and delivery of biomolecules for skin regeneration. Mater. Sci. Eng. C Mater. Biol. Appl..

[102] Sudheesh Kumar P.T., Lakshmanan V.K., Raj M., Biswas R., Hiroshi T., Nair S.V., Jayakumar R. (2013). Evaluation of wound healing potential of β-chitin hydrogel/nano zinc oxide composite bandage. Pharm. Res..

[103] Chigurupati S., Mughal M.R., Okun E., Das S., Kumar A., McCaffery M., Seal S., Mattson M.P. (2013). Effects of cerium oxide nanoparticles on the growth of keratinocytes, fibroblasts and vascular endothelial cells in cutaneous wound healing. Biomaterials.

[104] Chen S.A., Chen H.M., Yao Y.D., Hung C.F., Tu C.S., Liang Y.J. (2012). Topical treatment with anti-oxidants and Au nanoparticles promote healing of diabetic wound through receptor for advance glycation end-products. Eur. J. Pharm. Sci..

[105] Zhou Z., Joslin S., Dellinger A., Ehrich M., Brooks B., Ren Q., Rodeck U., Lenk R., Kepley C.L. (2010). A novel class of compounds with cutaneous wound healing properties. J. Biomed. Nanotechnol..

[106] Shahnawaz Khan M., Abdelhamid H.N., Wu H.F. (2015). Near infrared (NIR) laser mediated surface activation of graphene oxide nanoflakes for efficient antibacterial, antifungal and wound healing treatment. Colloids Surf. B Biointerfaces.

[107] Barui A.K., Veeriah V., Mukherjee S., Manna J., Patel A.K., Patra S., Pal K., Murali S., Rana R.K., Chatterjee S. (2012). Zinc oxide nanoflowers make new blood vessels. Nanoscale.

[108] Xie Z., Paras C.B., Weng H., Punnakitikashem P., Su L.C., Vu K., Tang L., Yang J., Nguyen K.T. (2013). Dual growth factor releasing multi-functional nanofibers for wound healing. Acta Biomater..

[109] Ma K., Liao S., He L., Lu J., Ramakrishna S., Chan C.K. (2011). Effects of nanofiber/stem cell composite on wound healing in acute full-thickness skin wounds. Tissue Eng. Part A.

[110] Mao C., Chen X., Miao G., Lin C. (2015). Angiogenesis stimulated by novel nanoscale bioactive glasses. Biomed. Mater..

[111] Kwon M.J., An S., Choi S., Nam K., Jung H.S., Yoon C.S., Ko J.H., Jun H.J., Kim T.K., Jung S.J. (2012). Effective healing of diabetic skin wounds by using nonviral gene therapy based on minicircle vascular endothelial growth factor DNA and a cationic dendrimer. J. Gene Med..

[112] Castangia I., Nácher A., Caddeo C., Valenti D., Fadda A.M., Díez-Sales O., Ruiz-Saurí A., Manconi M. (2014). Fabrication of quercetin and curcumin bionanovesicles for the prevention and rapid regeneration of full-thickness skin defects on mice. Acta Biomater..

[113] Tiwari M., Narayanan K., Thakar M.B., Jagani H.V., Venkata Rao J. (2014). Biosynthesis and wound healing activity of copper nanoparticles. IET Nanobiotechnol..

[114] Pal S., Tak Y.K., Song J.M. (2007). Does the antibacterial activity of silver nanoparticles depend on the shape of the nanoparticle? A study of the Gram-negative bacterium Escherichia coli. Appl. Environ. Microbiol..

[115] Lu Z., Yu D., Nie F., Wang Y., Chong Y. (2023). Iron Nanoparticles Open Up New Directions for Promoting Healing in Chronic Wounds in the Context of Bacterial Infection. Pharmaceutics.

[116] Yang M., Zhang J., Shi W., Zhang J., Tao C. (2022). Recent advances in metal–organic frameworks and their composites for the phototherapy of skin wounds. J. Mater. Chem. B.

[117] Xiong Y., Feng Q., Lu L., Qiu X., Knoedler S., Panayi A.C., Jiang D., Rinkevich Y., Lin Z., Mi B. (2024). Metal-Organic Frameworks and Their Composites for Chronic Wound Healing: From Bench to Bedside. Adv. Mater..

[118] Nascimento E.G.d., Sampaio T.B.M., Medeiros A.C., Azevedo E.P.d. (2009). Evaluation of chitosan gel with 1% silver sulfadiazine as an alternative for burn wound treatment in rats. Acta Cir. Bras..

[119] Hamdan S., Pastar I., Drakulich S., Dikici E., Tomic-Canic M., Deo S., Daunert S. (2017). Nanotechnology-Driven Therapeutic Interventions in Wound Healing: Potential Uses and Applications. ACS Cent. Sci..

[120] Samaha R., Othman A., El-Sherbiny I., Amer M., Elhusseini F., ElMissiry M. (2017). Topical Nitric oxide in nanoformulation enhanced wound healing in experimental diabetes in mice. Res. J. Pharm. Biol. Chem. Sci..

[121] Mofazzal Jahromi M.A., Sahandi Zangabad P., Moosavi Basri S.M., Sahandi Zangabad K., Ghamarypour A., Aref A.R., Karimi M., Hamblin M.R. (2018). Nanomedicine and advanced technologies for burns: Preventing infection and facilitating wound healing. Adv. Drug Deliv. Rev..

[122] Sharma A., Kumar A., Li C., Sharma R.K., Swihart M.T. (2020). Microencapsulated UV filter@ZIF-8 based sunscreens for broad spectrum UV protection. RSC Adv..

[123] Yadav A., Sharma A., Sharma R.K. (2019). Mesoporous iron gallate nanocomplex for adsorption and degradation of organic dyes. Colloids Surf. A Physicochem. Eng. Asp..

[124] Sharma A., Yadav A., Cwiklinski K., Quaye E., Aalinkeel R., Mahajan S.D., Schwartz S.A., Sharma R.K. (2019). In-vitro studies of curcumin encapsulated mesoporous Fe-Phenanthroline nanocluster for reduction of amyloid β plaque. J. Drug Deliv. Sci. Technol..

[125] Welsh J.A., Goberdhan D.C.I., O’Driscoll L., Buzas E.I., Blenkiron C., Bussolati B., Cai H., Di Vizio D., Driedonks T.A.P., Erdbrügger U. (2024). Minimal information for studies of extracellular vesicles (MISEV2023): From basic to advanced approaches. J. Extracell. Vesicles.

[126] Ghatak S., Khanna S., Roy S., Thirunavukkarasu M., Pradeep S.R., Wulff B.C., El Masry M.S., Sharma A., Palakurti R., Ghosh N. (2023). Driving adult tissue repair via re-engagement of a pathway required for fetal healing. Mol. Ther..

[127] Prasai A., Jay J.W., Jupiter D., Wolf S.E., El Ayadi A. (2022). Role of Exosomes in Dermal Wound Healing: A Systematic Review. J. Investig. Dermatol..

[128] Dai W., Dong Y., Han T., Wang J., Gao B., Guo H., Xu F., Li J., Ma Y. (2022). Microenvironmental cue-regulated exosomes as therapeutic strategies for improving chronic wound healing. NPG Asia Mater..

[129] Zhao H., Li Z., Wang Y., Zhou K., Li H., Bi S., Wang Y., Wu W., Huang Y., Peng B. (2023). Bioengineered MSC-derived exosomes in skin wound repair and regeneration. Front. Cell Dev. Biol..

[130] Riha S.M., Maarof M., Fauzi M.B. (2021). Synergistic Effect of Biomaterial and Stem Cell for Skin Tissue Engineering in Cutaneous Wound Healing: A Concise Review. Polymers.

[131] Cao J., Wu B., Yuan P., Liu Y., Hu C. (2023). Rational Design of Multifunctional Hydrogels for Wound Repair. J. Funct. Biomater..

[132] Zhang W., Liu W., Long L., He S., Wang Z., Liu Y., Yang L., Chen N., Hu C., Wang Y. (2023). Responsive multifunctional hydrogels emulating the chronic wounds healing cascade for skin repair. J. Control. Release.

[133] Zhou Y., Zhang X.-L., Lu S.-T., Zhang N.-Y., Zhang H.-J., Zhang J., Zhang J. (2022). Human adipose-derived mesenchymal stem cells-derived exosomes encapsulated in pluronic F127 hydrogel promote wound healing and regeneration. Stem Cell Res. Ther..

[134] Hao D., Swindell H.S., Ramasubramanian L., Liu R., Lam K.S., Farmer D.L., Wang A. (2020). Extracellular Matrix Mimicking Nanofibrous Scaffolds Modified with Mesenchymal Stem Cell-Derived Extracellular Vesicles for Improved Vascularization. Front. Bioeng. Biotechnol..

[135] Farabi B., Roster K., Hirani R., Tepper K., Atak M.F., Safai B. (2024). The Efficacy of Stem Cells in Wound Healing: A Systematic Review. Int. J. Mol. Sci..

[136] Pal D., Ghatak S., Singh K., Abouhashem A.S., Kumar M., El Masry M.S., Mohanty S.K., Palakurti R., Rustagi Y., Tabasum S. (2023). Identification of a physiologic vasculogenic fibroblast state to achieve tissue repair. Nat. Commun..

[137] Srivastava R., Singh K., Abouhashem A.S., Kumar M., Kacar S., Verma S.S., Mohanty S.K., Sinha M., Ghatak S., Xuan Y. (2023). Human fetal dermal fibroblast-myeloid cell diversity is characterized by dominance of pro-healing Annexin1-FPR1 signaling. iScience.

[138] Long C., Wang J., Gan W., Qin X., Yang R., Chen X. (2022). Therapeutic potential of exosomes from adipose-derived stem cells in chronic wound healing. Front. Surg..

[139] Guda P.R., Sharma A., Anthony A.J., ElMasry M.S., Couse A.D., Ghatak P.D., Das A., Timsina L., Trinidad J.C., Roy S. (2023). Nanoscopic and Functional Characterization of Keratinocyte-Originating Exosomes in the Wound Fluid of Non-Diabetic and Diabetic Chronic Wound Patients. Nano Today.

[140] Brown B.A., Guda P.R., Zeng X., Anthony A., Couse A., Barnes L.F., Sharon E.M., Trinidad J.C., Sen C.K., Jarrold M.F. (2022). Analysis of Keratinocytic Exosomes from Diabetic and Nondiabetic Mice by Charge Detection Mass Spectrometry. Anal. Chem..

[141] Zhou X., Brown B.A., Siegel A.P., El Masry M.S., Zeng X., Song W., Das A., Khandelwal P., Clark A., Singh K. (2020). Exosome-Mediated Crosstalk between Keratinocytes and Macrophages in Cutaneous Wound Healing. ACS Nano.

[142] Pi L., Yang L., Fang B.R., Meng X.X., Qian L. (2022). LncRNA MALAT1 from human adipose-derived stem cell exosomes accelerates wound healing via miR-378a/FGF2 axis. Regen. Med..

[143] Tan K., Mo H., Guo L., Wang B. (2022). MALAT1 accelerates proliferation and inflammation and suppresses apoptosis of endometrial stromal cells via the microRNA-142-3p/CXCR7 axis. Reprod. Biol..

[144] Narauskaitė D., Vydmantaitė G., Rusteikaitė J., Sampath R., Rudaitytė A., Stašytė G., Aparicio Calvente M.I., Jekabsone A. (2021). Extracellular Vesicles in Skin Wound Healing. Pharmaceuticals.

[145] Li M., Wang T., Tian H., Wei G., Zhao L., Shi Y. (2019). Macrophage-derived exosomes accelerate wound healing through their anti-inflammation effects in a diabetic rat model. Artif. Cells Nanomed. Biotechnol..

[146] Han X., Wu P., Li L., Sahal H.M., Ji C., Zhang J., Wang Y., Wang Q., Qian H., Shi H. (2021). Exosomes derived from autologous dermal fibroblasts promote diabetic cutaneous wound healing through the Akt/β-catenin pathway. Cell Cycle.

[147] Xia W., Li M., Jiang X., Huang X., Gu S., Ye J., Zhu L., Hou M., Zan T. (2022). Young fibroblast-derived exosomal microRNA-125b transfers beneficial effects on aged cutaneous wound healing. J. Nanobiotechnol..

[148] Hu S., Li Z., Cores J., Huang K., Su T., Dinh P.U., Cheng K. (2019). Needle-Free Injection of Exosomes Derived from Human Dermal Fibroblast Spheroids Ameliorates Skin Photoaging. ACS Nano.

[149] Qie Y., Yuan H., von Roemeling C.A., Chen Y., Liu X., Shih K.D., Knight J.A., Tun H.W., Wharen R.E., Jiang W. (2016). Surface modification of nanoparticles enables selective evasion of phagocytic clearance by distinct macrophage phenotypes. Sci. Rep..

[150] Alvarez-Erviti L., Seow Y., Yin H., Betts C., Lakhal S., Wood M.J. (2011). Delivery of siRNA to the mouse brain by systemic injection of targeted exosomes. Nat. Biotechnol..

[151] Donoso-Quezada J., Ayala-Mar S., González-Valdez J. (2020). State-of-the-art exosome loading and functionalization techniques for enhanced therapeutics: A review. Crit. Rev. Biotechnol..

[152] Wang J., Li W., Zhang L., Ban L., Chen P., Du W., Feng X., Liu B.-F. (2017). Chemically Edited Exosomes with Dual Ligand Purified by Microfluidic Device for Active Targeted Drug Delivery to Tumor Cells. ACS Appl. Mater. Interfaces.

[153] Sousa de Almeida M., Susnik E., Drasler B., Taladriz-Blanco P., Petri-Fink A., Rothen-Rutishauser B. (2021). Understanding nanoparticle endocytosis to improve targeting strategies in nanomedicine. Chem. Soc. Rev..

[154] Lu J., Gao X., Wang S., He Y., Ma X., Zhang T., Liu X. (2023). Advanced strategies to evade the mononuclear phagocyte system clearance of nanomaterials. Exploration.

[155] Khona D.K., Roy S., Ghatak S., Huang K., Jagdale G., Baker L.A., Sen C.K. (2021). Ketoconazole resistant Candida albicans is sensitive to a wireless electroceutical wound care dressing. Bioelectrochemistry.

[156] Sousa P., Lopes B., Sousa A.C., Moreira A., Coelho A., Alvites R., Alves N., Geuna S., Maurício A.C. (2023). Advancements and Insights in Exosome-Based Therapies for Wound Healing: A Comprehensive Systematic Review (2018–June 2023). Biomedicines.

[157] Zeng H., Guo S., Ren X., Wu Z., Liu S., Yao X. (2023). Current Strategies for Exosome Cargo Loading and Targeting Delivery. Cells.

[158] Sharma A., Yadav A., Nandy A., Ghatak S. (2024). Insight into the Functional Dynamics and Challenges of Exosomes in Pharmaceutical Innovation and Precision Medicine. Pharmaceutics.

[159] Yadav A., Xuan Y., Sen C.K., Ghatak S. (2024). Standardized Reporting of Research on Exosomes to Ensure Rigor and Reproducibility. Adv. Wound Care.

[160] Li J., Ghatak S., El Masry M.S., Das A., Liu Y., Roy S., Lee R.J., Sen C.K. (2018). Topical Lyophilized Targeted Lipid Nanoparticles in the Restoration of Skin Barrier Function following Burn Wound. Mol. Ther..

[161] Koh H.B., Kim H.J., Kang S.W., Yoo T.H. (2023). Exosome-Based Drug Delivery: Translation from Bench to Clinic. Pharmaceutics.

[162] Kalluri R., LeBleu V.S. (2020). The biology, function, and biomedical applications of exosomes. Science.

[163] Ye H., Wang F., Xu G., Shu F., Fan K., Wang D. (2023). Advancements in engineered exosomes for wound repair: Current research and future perspectives. Front. Bioeng. Biotechnol..

[164] Li Z., Xuan Y., Ghatak S., Guda P.R., Roy S., Sen C.K. (2022). Modeling the gene delivery process of the needle array-based tissue nanotransfection. Nano Res..

[165] Xuan Y., Wang C., Ghatak S., Sen C.K. (2024). Tissue Nanotransfection Silicon Chip and Related Electroporation-Based Technologies for In Vivo Tissue Reprogramming. Nanomaterials.

[166] Weng Z., Zhang B., Wu C., Yu F., Han B., Li B., Li L. (2021). Therapeutic roles of mesenchymal stem cell-derived extracellular vesicles in cancer. J. Hematol. Oncol..

[167] Parada N., Romero-Trujillo A., Georges N., Alcayaga-Miranda F. (2021). Camouflage strategies for therapeutic exosomes evasion from phagocytosis. J. Adv. Res..

[168] Liang Y., Duan L., Lu J., Xia J. (2021). Engineering exosomes for targeted drug delivery. Theranostics.

[169] Damasceno P.K.F., de Santana T.A., Santos G.C., Orge I.D., Silva D.N., Albuquerque J.F., Golinelli G., Grisendi G., Pinelli M., Ribeiro Dos Santos R. (2020). Genetic Engineering as a Strategy to Improve the Therapeutic Efficacy of Mesenchymal Stem/Stromal Cells in Regenerative Medicine. Front. Cell Dev. Biol..

[170] Si C., Gao J., Ma X. (2024). Engineered exosomes in emerging cell-free therapy. Front. Oncol..

[171] Li T., Li X., Han G., Liang M., Yang Z., Zhang C., Huang S., Tai S., Yu S. (2022). The Therapeutic Potential and Clinical Significance of Exosomes as Carriers of Drug Delivery System. Pharmaceutics.

[172] Antes T.J., Middleton R.C., Luther K.M., Ijichi T., Peck K.A., Liu W.J., Valle J., Echavez A.K., Marbán E. (2018). Targeting extracellular vesicles to injured tissue using membrane cloaking and surface display. J. Nanobiotechnol..

[173] Sharma V., Mukhopadhyay C.D. (2024). Exosome as drug delivery system: Current advancements. Extracell. Vesicle.

[174] Ferreira D., Moreira J.N., Rodrigues L.R. (2022). New advances in exosome-based targeted drug delivery systems. Crit. Rev. Oncol. Hematol..

[175] Joshi B.S., de Beer M.A., Giepmans B.N.G., Zuhorn I.S. (2020). Endocytosis of Extracellular Vesicles and Release of Their Cargo from Endosomes. ACS Nano.

[176] Canton I., Battaglia G. (2012). Endocytosis at the nanoscale. Chem. Soc. Rev..

[177] Zhang S., Gao H., Bao G. (2015). Physical Principles of Nanoparticle Cellular Endocytosis. ACS Nano.

[178] Rodriguez P.L., Harada T., Christian D.A., Pantano D.A., Tsai R.K., Discher D.E. (2013). Minimal “Self” peptides that inhibit phagocytic clearance and enhance delivery of nanoparticles. Science.

[179] Kuang L., Zhang C., Li B., Deng H., Chen R., Li G. (2023). Human Keratinocyte-Derived Exosomal MALAT1 Promotes Diabetic Wound Healing by Upregulating MFGE8 via microRNA-1914-3p. Int. J. Nanomed..

[180] Glady A., Vandebroek A., Yasui M. (2021). Human keratinocyte-derived extracellular vesicles activate the MAPKinase pathway and promote cell migration and proliferation in vitro. Inflamm. Regen..

[181] Belvedere R., Pessolano E., Porta A., Tosco A., Parente L., Petrella F., Perretti M., Petrella A. (2020). Mesoglycan induces the secretion of microvesicles by keratinocytes able to activate human fibroblasts and endothelial cells: A novel mechanism in skin wound healing. Eur. J. Pharmacol..

[182] Liu K., Luo X., Lv Z.-Y., Zhang Y.-J., Meng Z., Li J., Meng C.-X., Qiang H.-F., Hou C.-Y., Hou L. (2022). Macrophage-Derived Exosomes Promote Bone Mesenchymal Stem Cells Towards Osteoblastic Fate Through microRNA-21a-5p. Front. Bioeng. Biotechnol..

[183] Gangadaran P., Rajendran R.L., Oh J.M., Hong C.M., Jeong S.Y., Lee S.-W., Lee J., Ahn B.-C. (2020). Extracellular vesicles derived from macrophage promote angiogenesis In vitro and accelerate new vasculature formation In vivo. Exp. Cell Res..

[184] Xiong Y., Chen L., Liu P., Yu T., Lin C., Yan C., Hu Y., Zhou W., Sun Y., Panayi A.C. (2022). All-in-One: Multifunctional Hydrogel Accelerates Oxidative Diabetic Wound Healing through Timed-Release of Exosome and Fibroblast Growth Factor. Small.

[185] Kwak G., Cheng J., Kim H., Song S., Lee S.J., Yang Y., Jeong J.H., Lee J.E., Messersmith P.B., Kim S.H. (2022). Sustained Exosome-Guided Macrophage Polarization Using Hydrolytically Degradable PEG Hydrogels for Cutaneous Wound Healing: Identification of Key Proteins and MiRNAs, and Sustained Release Formulation. Small.

[186] Lesniak A., Fenaroli F., Monopoli M.P., Åberg C., Dawson K.A., Salvati A. (2012). Effects of the presence or absence of a protein corona on silica nanoparticle uptake and impact on cells. ACS Nano.

[187] Suk J.S., Xu Q., Kim N., Hanes J., Ensign L.M. (2016). PEGylation as a strategy for improving nanoparticle-based drug and gene delivery. Adv. Drug Deliv. Rev..

[188] Shi D., Beasock D., Fessler A., Szebeni J., Ljubimova J.Y., Afonin K.A., Dobrovolskaia M.A. (2022). To PEGylate or not to PEGylate: Immunological properties of nanomedicine’s most popular component, polyethylene glycol and its alternatives. Adv. Drug Deliv. Rev..

[189] Cifuentes-Rius A., Desai A., Yuen D., Johnston A.P.R., Voelcker N.H. (2021). Inducing immune tolerance with dendritic cell-targeting nanomedicines. Nat. Nanotechnol..

[190] Grady C. (2015). Institutional Review Boards: Purpose and Challenges. Chest.

